# Proton Therapy for Stage IIA-B Seminoma: A New Standard of Care for Treating Retroperitoneal Nodes

**DOI:** 10.14338/IJPT-18-00001.1

**Published:** 2018-11-30

**Authors:** Richard Choo, Bret Kazemba, Christopher S. Choo, Scott C. Lester, Thomas Whitaker

**Affiliations:** 1 Department of Radiation Oncology, Mayo Clinic, Rochester, MN, USA; 2 Department of Internal Medicine, University of Minnesota, Minneapolis, MN, USA

**Keywords:** proton beam, stage II seminoma, retroperitoneal lymph nodes

## Abstract

Currently there has been no published report describing the use of proton beam therapy for stage II testicular seminoma. A 31-year-old man presenting with a right testicular mass and a 2.7-cm aortocaval lymph node received a diagnosis of stage IIB testicular seminoma. He was treated with scanning proton beam therapy, as a means of improving the therapeutic ratio of radiation therapy over conventionally used x-ray radiation therapy. The patient achieved a complete response and remained free of relapse at 15 months post proton beam therapy. The advantageous dose deposition characteristics of proton beam, allowing much lower radiation doses to normal tissues, should be exploited when radiation therapy is applied for stage II testicular seminoma or for an isolated retroperitoneal lymph node relapse of stage I disease initially managed with surveillance.

## Introduction

Testicular seminoma mainly affects young males between the ages of 20 and 45 years, and is highly curable with modern therapies. Over the last 3 decades, major concerns have been raised for potential late treatment-related morbidities, such as a secondary malignancy and an increased risk of cardiovascular disease. As a result, efforts in the management of stage I and II testicular seminoma have been focused on how to improve the therapeutic ratio by minimizing treatment-related toxicity without compromising disease-specific survival. These efforts have led to a steady de-escalation of treatment intensity over time, and surveillance alone with the reservation of radiation therapy or chemotherapy for a relapse has become the standard of care for stage I testicular seminoma.

For stage II testicular seminoma with retroperitoneal nodal metastasis, management options include radiation therapy and cisplatin-based combination chemotherapy. For a patient with retroperitoneal nodal metastasis ≤ 5 cm (ie, stage IIA and IIB), radiation therapy is a preferred treatment option because it has more favorable side effect profile than combination chemotherapy. When radiation therapy is considered for such patients, proton beam therapy can yield favorable dose deposition characteristics. The adoption of proton beam therapy is consistent with current efforts to continue improving the therapeutic ratio in patients with testicular seminoma, since it can provide a significant reduction in the dose to multiple normal organs that will likely translate into a decreased risk of late radiation-related morbidities, compared to conventional x-ray radiation therapy.

## Case Report

### Medical History

**Figure 1:**
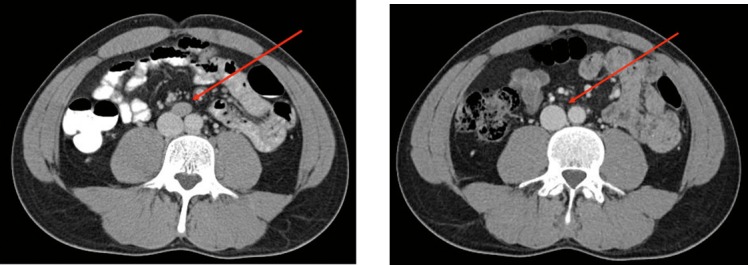
(A) Enlarged aortocaval lymph node at presentation. (B) Follow-up of the aortocaval lymph node at 15 months post proton beam therapy.

A 31-year-old white man, just married 5 months prior, presented with a nontender right testicular mass. A scrotal ultrasonography confirmed a hypoechoic mass in the right testicle. A computed tomography (CT) scan of chest was unremarkable. A CT scan of abdomen and pelvis revealed an enlarged aortocaval lymph node, measuring 1.5 × 1.9 × 2.7 cm (**Figure 1A**). α-Fetoprotein and β–human chorionic gonadotropin concentrations were normal. Two months later, he underwent a right radical orchiectomy. Pathology revealed a pure seminoma measuring 1.8 × 1.5 × 1.4 cm. Surgical margins including the spermatic cord margin were clear. There was no rete testis invasion or lymphovascular space involvement. The seminoma was staged IIB. He was advised to undergo definitive radiation therapy for his stage IIB seminoma.

### Radiation Therapy Plan: Proton versus Photon

The patient underwent a CT simulation with 2-mm-slice thickness in the supine position with a vacuum bag device (Civco Inc, Orange City, IA) for the lower legs. There were 2 clinical target volumes (CTVs). The first CTV (CTV1) was contoured around the aorta, inferior vena cava, and ipsilateral iliac vessels with a 7- to 10-mm expansion between the level of the mid-T11 vertebrae and the mid obturator foramen. CTV1 encompassed the periaortic, pericaval, aortocaval, and ipsilateral iliac lymph nodes. The second CTV (CTV2) was to deliver a boost dose to the region of the enlarged node, and contoured in a similar manner. The superior and inferior extent of CTV2 was delineated by using a 20-mm expansion from the involved node. A 5-mm margin (incorporating a 3-mm setup uncertainty and 3% range uncertainty) was then added to the CTV1 and CTV2 to generate optimization target volumes (OTV1 and OTV2) for a proton plan. The same margin was used to create planning target volumes (PTV1 and PTV2) for a comparison photon plan.

**Figure 2:**
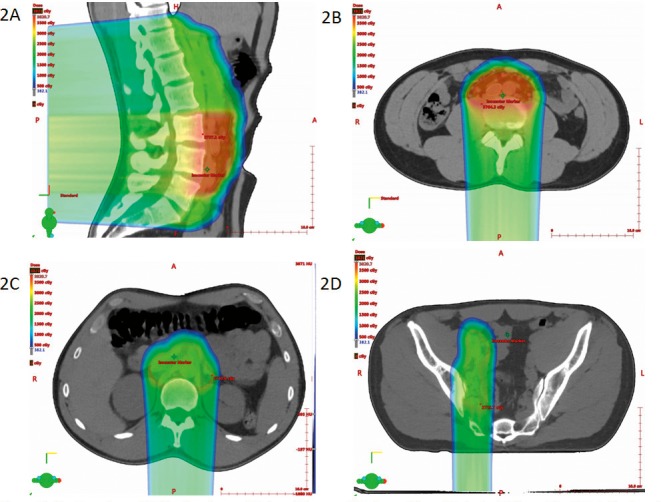
Proton dose distribution in sagittal and 3 axial views: (A) sagittal at mid line, (B) axial at the level of enlarged aortocaval lymph node, (C) axial at 13.4 cm superior to isocenter, and (D) axial at 8.5 cm inferior to isocenter.

A proton plan was created by using a single posterior scanning beam (**Figure 2A** through **2D**). A radiation dose of 25.5 Gy(RBE) in 17 fractions was prescribed to the OTV1. This was followed by a boost dose of 10 Gy(RBE) in 5 fractions to the OTV2. Orthogonal kilovoltage imaging was used daily before each fraction to verify alignment of the bony structures (especially the lumbar spine) in relation to the digitally reconstructed radiographs generated from the simulation CT images. In addition, verification CT scans were obtained weekly during the course of proton beam therapy to ensure that the prepared plan would provide adequate coverage of the CTVs and OTVs. No re-planning was needed as each verification scan confirmed satisfactory coverage of the CTVs and OTVs.

**Figure 3:**
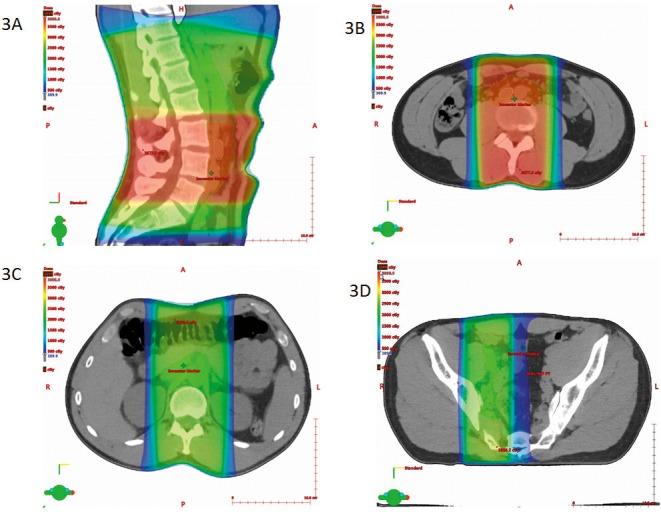
Photon dose distribution in sagittal and 3 axial views: (A) sagittal at mid line, (B) axial at the level of enlarged aortocaval lymph node, (C) axial at 13.4 cm superior to isocenter, and (D) axial at 8.5 cm inferior to isocenter.

A 3-dimentional conformal photon plan was prepared with 10-MV beams, using the aforementioned CTVs and PTVs, for comparison. Anterior-posterior and posterior-anterior fields were used with appropriate field weighing (**Figure 3A** through **3D**).

### Dose-Volume Histogram for the Targets and Organs at Risk: Proton versus Photon Plans

Both proton and photon plans were created by using identical target dose-volume histogram (DVH) objectives. These included 100% of the CTVs receiving 98% of the prescription dose, and 100% of the OTVs and PTVs receiving 95% of the prescription dose. The DVHs of the targets and organs at risk (OARs) were computed by using the Eclipse treatment planning system (Varian Medical Systems, Palo Alto, California) for both the proton and photon plans. Mean doses and percentage volumes of OAR receiving various doses were calculated and compared between the 2 plans.

Both plans provided adequate coverage of the CTVs as well as OTVs and PTVs (**Table 1**). **Table 2** depicts DVH metrics for OARs. The proton plan delivered significantly less doses to the normal tissues, particularly the anterior structures such as the liver, stomach, small bowel, and large bowel. The doses to the kidneys were also reduced with the proton plan.

**Table 1:** Dose-volume histogram for CTV1 and CTV2: proton versus photon.

	**CTV1**		**CTV2**	
	**Proton**	**Photon**	**Proton**	**Photon**
Mean, Gy	29.09^a^	29.67^a^	36.44	36.84
V107%, %	31.6^a^	39.5^a^	0	0
V100%, %	99.3	95.2	99.7	100
D98%, %	100.5	98.7	100.6	101.3
D95%, %	100.9	100.1	101	101.7
D2%, %	145^a^	146.6^a^	104.9	105.7

**Abbreviation**: CTV, clinical target volume.

a Owing to the inclusion of CTV2.

**Table 2:** Dose-volume histogram for organs at risk: proton versus photon.

	**Proton**	**Photon**
Heart		
Mean dose, Gy	0	0.56
V5Gy, %	0	0.2
V10Gy, %	0	0
Liver		
Mean dose, Gy	0.40	3.22
V5Gy, %	2.4	16.2
V10Gy, %	1.5	8.9
V15Gy, %	1	5.5
V20Gy, %	0.6	4.4
V25Gy, %	0.2	1.5
V30Gy, %	0	0
Stomach		
Mean dose, Gy	0.52	8.40
V5Gy, %	2.9	36.4
V10Gy, %	1.8	29.2
V15Gy, %	1.3	26
V20Gy, %	0.9	23.9
V25Gy, %	0.4	18.2
V30Gy, %	0	0
Large bowel		
Mean dose, Gy	0.48	8.03
V5Gy, %	3.2	29.4
V10Gy, %	1.3	25.8
V15Gy, %	0.6	23.8
V20Gy, %	0.2	22.5
V25Gy, %	0	19.5
V30Gy, %	0	0
Small bowel		
Mean dose, Gy	5.54	9.52
V5Gy, %	27.4	36
V10Gy, %	22.5	30.7
V15Gy, %	18.3	27.9
V20Gy, %	14.2	25.7
V25Gy, %	9.2	20.7
V30Gy, %	1.9	5.3
V35Gy, %	0.5	4
V37Gy, %	0.1	1
Rectum		
Mean dose, Gy	3.07	7.62
V5Gy, %	16.1	31
V10Gy, %	13	24.3
V15Gy, %	10.5	21.1
V20Gy, %	8	18.6
V25Gy, %	3.6	12.9
V30Gy, %	0	0
Pancreas		
Mean dose, Gy	16.42	24.61
V5Gy, %	84.6	98.8
V10Gy, %	71.5	96.1
V15Gy, %	57.9	94.1
V20Gy, %	44.2	91
V25Gy, %	27.3	73.2
V30Gy, %	0	0
Bladder		
Mean dose, Gy	4.91	9.39
V5Gy, %	24	38.4
V10Gy, %	20.6	33.1
V15Gy, %	17.9	29.9
V20Gy, %	14.9	26.4
V25Gy, %	6.5	19
V30Gy, %	0	0
Left testicle		
Mean dose, Gy	0	0.07
V5Gy, %	0	0
Left kidney		
Mean dose, Gy	3.04	7.07
V5Gy, %	16	30.8
V10Gy, %	13.4	24.4
V15Gy, %	11.4	19.9
V20Gy, %	8.4	17
V25Gy, %	1.2	5.5
V30Gy, %	0	0
Right kidney		
Mean dose, Gy	3.10	8.12
V5Gy, %	17.7	36.5
V10Gy, %	13.6	27.7
V15Gy, %	10.5	21.8
V20Gy, %	6.3	17.8
V25Gy, %	1	7.1
V30Gy, %	0	0
Spinal cord		
Max dose, Gy	21.36	28.16
Mean dose, Gy	9.46	14.57
V5Gy, %	49.6	57.9
V10Gy, %	48.6	53.4
V15Gy, %	47.5	50.1
V20Gy, %	8	49.5
V25Gy, %	0	48.1
V30Gy, %	0	0

**Abbreviation**: Max, maximum.

### Follow-up

The patient completed proton beam therapy without any treatment interruption and experienced only self-limiting minimal nausea (grade 1, based on Common Terminology Criteria for Adverse Events v4.0). At the 15-month follow-up evaluation, a CT scan of the chest, abdomen, and pelvis showed that the enlarged lymph node remained completely resolved (**Figure 1B**) with no evidence of nodal or distant metastasis. His laboratory results including β–human chorionic gonadotropin and α-fetoprotein, and physical examination, were also normal. He was considered in complete remission. His wife was 12 weeks' pregnant, and they conceived without using sperms banked before the radiation therapy.

## Discussion

Radiation therapy with the reservation of combination chemotherapy for the salvage of post–radiation therapy relapse has been the mainstay for the management of stage IIA-IIB testicular seminoma. The CTV includes the periaortic, pericaval, aortocaval, and ipsilateral iliac lymph nodes, and the irradiation field typically encompasses the region from the superior endplate of the T11 vertebrae down to the middle of the obturator foramen.

Given that patients with testicular seminoma usually receive a diagnosis at a young age and live long after treatment with a very high cure rate, the major concern for these patients has been the potential late sequelae of treatment, such as radiation-related secondary malignancies. Therefore, recent efforts have focused on reducing treatment-related toxicity while preserving a high rate of cure. Such efforts have included the decrease in radiation dose and field size as well as the adoption of surveillance as the preferred management strategy for stage I testicular seminoma. Another such strategy is the utilization of proton beam radiation therapy instead of conventional x-ray radiation therapy, when radiation therapy is indicated, given the more favorable dose deposition characteristics of proton beam therapy. There has been a single case report describing the utilization of proton beam therapy for a patient who experienced a relapse in a pericaval lymph node after being initially managed with surveillance [1]. A few other reports assessing proton beam therapy were dosimetric studies describing the rationales for using proton beam therapy in patients with stage I and II seminoma [2–4].

This case report illustrates that proton beam therapy provides adequate coverage of CTVs while significantly limiting the collateral dose to the surrounding normal organs. This dose reduction should translate into less acute side effects and reduced risk of late complications such as secondary malignancies. Potential acute side effects of radiation therapy include nausea, diarrhea, fatigue, and decreased blood counts. Theses side effects can be reduced significantly with proton beam therapy (in particular, with a single posterior beam arrangement), as it allows a significant decrease in the dose to normal organs such as the liver, stomach, small bowel, large bowel, and pancreas. In our case, V5Gy (%) to V25Gy (%) as well as mean doses of all the gastrointestinal organs, both kidneys, and bladder were reduced with proton beam therapy. Expected decreases in gastrointestinal side effects and radiation therapy–related lethargy portend a less adverse impact on quality of life and a higher likelihood of maintaining occupational or academic productivity.

One of the most concerning late side effects of radiation therapy is radiation-induced secondary cancers. Several studies evaluated and quantified this risk. Travis et al [5] reported that for patients with a diagnosis of seminoma at the age of 35 years, the cumulative risk of solid cancer 40 years later was 36% compared with an expected 23% for the general population; further, about 64% of solid tumors occurred within the irradiated field. As demonstrated in this case report, proton beam therapy can significantly reduce the dose to adjacent organs including the stomach, pancreas, small bowel, large bowel, rectum, and bladder. This dramatic dose reduction achieved with proton beam therapy should translate into lower rates of late radiation complications, including second malignancies, in seminoma survivors.

Other advantages of proton beam therapy include a reduced dose to the heart, which should translate into a decreased risk of cardiovalvular, cardiomuscular, and cardiovascular toxicity. Another advantage is a potential to decrease the dose to the remaining testis. Given that fertility issue can be a major concern for young men and that the testicular germinal epithelium is highly sensitive to radiation, reducing a dose to the remaining testis is an important aspect of overall treatment strategy. Since the proton beam penumbra in the lateral direction is smaller than that of conventional x-ray, it can reduce the dose to the remaining testis, which can translate into better preservation of spermatogenesis.

In conclusion, this case report presents a clinical and dosimetric rationale for using proton beam therapy for a patient with stage IIB seminoma. Proton beam radiation therapy improves the therapeutic ratio of radiation therapy with a notable reduction in the dose to multiple normal organs that should translate into reductions in the acute and late side effects of radiation therapy. Thus, when radiation therapy is indicated for patients with stage IIA-IIB, proton beam therapy should be strongly considered as a standard of care to maximize survival and quality of life.
